# Dynamic Light Scattering of Biopharmaceutics—Can Analytical Performance Be Enhanced by Laser Power?

**DOI:** 10.3390/pharmaceutics10030094

**Published:** 2018-07-17

**Authors:** Simone Aleandri, Andrea Vaccaro, Ricardo Armenta, Andreas Charles Völker, Martin Kuentz

**Affiliations:** 1 Institute of Pharma Technology, University of Applied Sciences and Arts Northwestern Switzerland, Gründenstrasse 40, 4132 Muttenz, Switzerland; simone.aleandri@fhnw.ch; 2 LS Instruments, Passage du Cardinal 1, 1700 Fribourg, Switzerland; andrea.vaccaro@lsinstruments.ch (A.V.); ricardo.armenta@lsinstruments.ch (R.A.); charles.voelker@lsinstruments.ch (A.C.V.)

**Keywords:** protein formulation, protein aggregation, dynamic light scattering (DSL), laser power, lysozyme

## Abstract

Background: Dynamic light scattering (DLS) is an important tool to characterize colloidal systems and adequate sizing is particularly critical in the field of protein formulations. Among the different factors that can influence the measurement result, the effect of laser power has so far not been studied thoroughly. Methods: The sensitivity of a DLS instrument was first considered on a theoretical level, followed by experiments using DLS instruments, equipped with two different lasers of (nominal) 45 mW, and 100 mW, respectively. This work analyzes dilute colloidal dispersions of lysozyme as model protein. Results: Theoretical findings agreed with experiments in that only enhanced laser power of 100 mW laser allowed measuring a 0.1 mg/mL protein dispersion in a reliable manner. Results confirmed the usefulness of the presented theoretical considerations in improving a general understanding of the limiting factors in DLS. Conclusions: Laser power is a critical aspect regarding adequate colloidal analysis by DLS. Practical guidance is provided to help scientists specifically with measuring dilute samples to choose both an optimal instrument configuration as well as a robust experimental procedure.

## 1. Introduction

**Figure 1:**
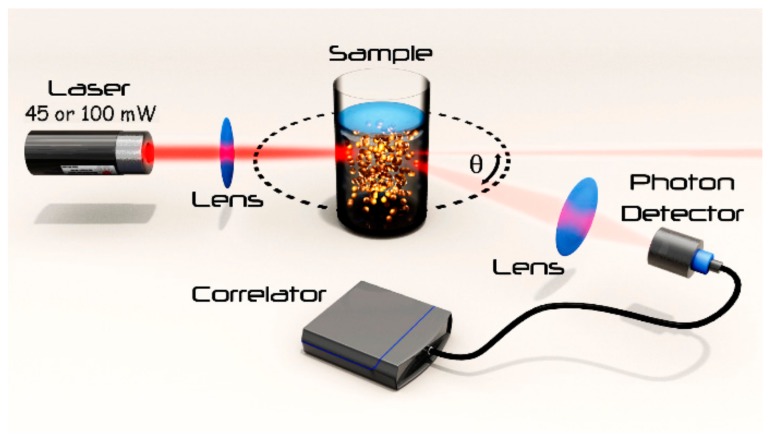
Depiction of the dynamic light scattering (DLS) apparatus with two different lasers of nominal powers 45 mW, and 100 mW, respectively. The sample is only schematically depicted to visualize the measurement principle.

Dynamic light scattering (DLS) has become an indispensable technique of size determination for diverse materials: from sub-micron particles to protein formulations [1]. The instruments on the market are generally user-friendly with software interfaces that, however, do not always give an indication of the quality of the generated data [2,3]. DLS is non-invasive, requires little sample preparation as well as small sample amounts [2]. Especially the latter aspect is critical for the development of biopharmaceutical products. The number of approved biotechnology medicines has greatly increased over the last two decades, and many new products are either in human clinical trials or under review by regulatory agencies [4]. A substantial number of these biological substances are proteins, which span from comparatively small biopharmaceuticals to rather large therapeutic antibodies [5], and their development comes with specific technical hurdles such as aggregation. Without stabilizers, such as surfactants, proteins tend to agglomerate in solution [6], which is a quality concern as it may affect safety as well as efficacy and therefore represents a significant barrier to product development [7]. Therefore, analytical methods to study the various types of protein aggregation constitute a research field in their own right, with the objective to offer characterization of protein size throughout the whole product lifecycle [8]. DLS is among the most important analytical techniques in this domain, since rapid measurement makes it possible to conduct high-throughput testing [9,10]. It is known that sample preparation method, storage conditions, ionic strength, and pH can influence the size and quality of proteins in a liquid formulation [11], and there is typically more than one analytical method used to detect subtle changes in the state of a protein [12]. A challenging aspect of particle analysis by DLS is the choice of the instrumental configuration as well as the definition of the experimental protocol. The quality of a measurement depends on instrument hardware, as well on the properties of the sample, such as size, contrast, concentration, and the presence of impurities. General guidelines are unfortunately still missing, and could aid formulation scientists with both instrument selection and definition of optimal experimental conditions [13]. One well-known problem of DLS that occurs in concentrated samples is multiple scattering, in which a photon scattered by one particle may be scattered again by another particle before reaching the detector. This results in artificially shorter decay times in the correlation function, inaccurately leading to smaller size results [14]. Multiple light scattering effects can be suppressed, for example, by implementing cross-correlation detection schemes [15], and more recently by the adoption of modulated 3D Cross-Correlation technology [16]. Opposite to the DLS challenge of multiple light scattering at high concentration is the analytical regime of very dilute samples, where there is not enough scattered light available for proper analysis. Several parameters of the instrumental setup [17] (such as scattering volume, angle of detection, laser power and detector sensitivity) or material properties [18] (molecular weight, compactness and shape) can influence the quality of the measurement. Specifically, protein samples with a size below 10 nm are difficult to analyze by DLS in dilute solutions [19]. Among the different factors that influence DLS analytics of dilute solutions, laser power and its implications on instrument sensitivity has not been studied well. The present work addresses this research gap by an initial set of theoretical considerations on the measurement, followed by their experimental verification with DLS-sizing measurements on the model protein lysozyme, performed in two different instruments equipped with lasers of either 45 mW, or 100 mW nominal power (see Figure 1). Even though the study focus is on the sizing of protein formulations, our findings are of much wider relevance for any DLS measurement of dilute colloids.

## 2. Theoretical Aspects: Scattering by Small Optically Soft Particles

As depicted in Figure 1, in a static light scattering experiment, a monochromatic, time-coherent light beam of wavelength *λ* (as typically emitted by lasers) impinges on a sample. The light reemitted, i.e., “scattered” by a sample is then collected at a certain angle *θ* with respect to the incident beam. If we consider a suspension of particles whose typical size, *R* is well below *λ*, i.e., that fulfills the so-called Rayleigh approximation [20], the time-averaged scattered intensity, *I*, can be expressed in terms of the particle’s molar mass *M*, and the mass concentration, *w*, as in the following Equation (1). It must be noted that the above considerations hold for a monodisperse particle suspension.

(1) $$I=I_{0}AwKM+I_{s}$$

The instrument sensitivity *A* is an instrumental constant that measures the sensitivity of the light scattering instrument used, and *K* is the sample contrast that mainly depends on the particle refractive index relative to that of the solvent. Moreover, *I*_0_ and *I_s_* are the light intensity of the laser and the light scattered by the pure solvent, respectively. In the present work the word “intensity” is used interchangeably for light irradiances and powers. Given that the detector area is fixed, this has no effect on the calculation other than the inclusion of the detector area into the unit measure of the optical constant *A*. The above Equation (1) can be recast as in Equation (2), by introducing the Rayleigh ratio (ℜ).

(2) $$I=I_{0}A\left[\Delta ℜ+{ℜ}_{s}\right]$$

Here, ℜ*_s_* represents the Rayleigh ratio of the solvent and Δℜ is the excess Rayleigh ratio (defined below in Equation (3)). The Rayleigh ratio is defined as the scattered intensity per unit incident intensity, unit solid angle, and unit scattering volume. The importance of the Rayleigh ratio lies in the fact that its value depends only on the thermodynamic state of the solvent and not on any instrumental detail.

(3) Δℜ≡wKM

When we consider the instantaneous value of the measured scattering intensity *I*(*t*) a DLS experiment is performed by computing its time correlation function as reported in Equation (4).

(4) $$g\left(\tau \right)\equiv \frac{\langle I\left(t\right)I(t+\tau )\rangle }{\langle I{\left(t\right)}^{2}\rangle }$$

where angle brackets denote a time average over *t*. Useful information about the particle size can be extracted in cases where particles undergo Brownian motion. Assuming no inter-particle interaction is present, *g*(*τ*) can be expressed as in Equation (5).

(5) $$g\left(\tau \right)=1+\beta \exp\left(-2q^{2}D\tau \right)$$

where *β* is the so-called intercept, *D* the particles’ diffusion coefficient and *q* is the module of the scattering vector that can be expressed in terms of the scattering angle (*θ*) as in Equation (6) where *n_s_* is the refractive index of the solvent.

(6) $$q=\frac{4\pi }{\lambda }n_{s}\sin(\theta /2)$$

It can be seen that by measuring the intensity correlation function of the scattered light intensity one can infer *D* of the suspended particles. If now the Stokes-Einstein equation is applied, we can define the hydrodynamic radius *R_h_*, which is related to the diffusion coefficient by Equation (7).

(7) $$R_{h}=\frac{k_{B}T}{6\pi \eta D}$$

where *k_B_* is the Boltzmann constant, *T* the system temperature, and *η* is the solvent viscosity. We have thus defined a quantity *R_h_* with the properties of a particle size and that can be visualized as the radius of an ideal colloidal sphere that (in the given experimental conditions) has a diffusion coefficient equal to that of the colloidal particles in the measurement sample. This quantity can be measured in a DLS experiment.

## 3. Materials and Methods

### 3.1. Sample Preparations

Lysozyme (purchased from Sigma Aldrich, Saint Louis, MO, USA) samples were prepared with different concentrations (from 5 to 0.1 mg/mL) in 0.1 M Na acetate buffer solution at pH 4.2. The samples were filtered using a 20 nm Anatop^®^ filter from Sigma Aldrich. Optical glass cuvettes (10 × 10 mm) were rinsed several times (at least five) with the filtered lysozyme dispersion and finally filled with the same solution under laminar flow hood to avoid dust contamination. The toluene solution used to evaluate the instrument constant *A* was filtered through the 20 nm Anatop filter and processed as described for lysozyme. 

### 3.2. Light Scattering Instruments

Two NanoLab 3D (LS instruments, Freiburg, Switzerland) were employed for the DLS analysis. They were equipped with a 660 nm, 100 mW or alternatively 45 mW at 685 nm, vertically polarized laser, and the detector at an angle of 90° with respect to the incident beam as depicted in Figure 1. The viscosity of the buffer at 25 °C (1.18 mPas) was used as solvent viscosity. The solvent viscosity was measured directly by the DLS methods. Briefly, the hydrodynamic radius of polystyrene nanoparticles (Megsphere, Pasadena, CA, USA) of 200 nm in diameter was measured in water (solvent with known viscosity). The obtained size value was then used as input during a measurement of the same particles, used as tracers in a buffer with particle suspension. According to the Stokes-Einstein’s equation (Equation (7)), one can measure the viscosity of the solvent of a particle suspension if the hydrodynamic radius *R_h_* of a tracer particle is known. Measurements were done in triplicate in auto correlation mode and the obtained values are reported as an average ± standard deviation (STDV). Each measurement was performed with a duration of 300 or 1000 s in case of the most diluted sample (0.1 mg/mL) with the laser intensity set at 100%. For the fitting of the correlation function, third-order cumulant fits were performed with the first channel index and the decay factor being 15 and 0.7, respectively. The scaled count rates were obtained from the ratio between raw count rates and the estimated incident laser power. Taking into account either the losses in fiber coupling and reflections, the estimated incident intensity can be considered to be around 70 mW.

## 4. Results and Discussion

### 4.1. Theoretical Sensitivity of a Dynamic Light Scattering (DLS) Instrument

To study the effect of laser power on the sensitivity of DLS analytics, one should first note that, due to electronic noise, the detector will exhibit so-called dark counts, which results in a detected background light intensity, *I*_DC_. Keeping that in mind, two regimes may be observed in evaluating the sensitivity of an instrument.

In the first case, the signal originating from the scattering by the solvent at the maximum available laser power is not substantially larger than that of the detector dark counts. The limit of measurability is here set by imposing that the signal from the particles be sufficiently larger than the dark counts signal. The larger the contrast and/or the molar mass *M*, the smaller the minimum measurable particle concentration *w*_min_. This can be expressed by the following Equation (8).

(8) $$w_{\min}=\frac{I_{\mathrm{DC}}}{KM}\frac{1}{I_{\max}A}$$

where *I*_max_ is the maximum incident laser power obtainable by the DLS instrument at hand, *K* is the sample contrast which mainly depends on the particle refractive index relative to that of the solvent and *A* is the instrument sensitivity constant previously introduced. Therefore, to achieve a more sensitive instrument, i.e., lower $w_{\min}$, one would need to lower the dark counts, increase the instrument sensitivity *A*, or increase the maximum laser power. 

An alternative case is given if the scattering signal by the solvent is substantially larger than that of the detector dark counts at the maximum available laser power. The limit in measurability with DLS is here dictated by the ratio of the signal arising from the particles to that from the solvent, according to Equation (9):

(9) $$w_{\min}=\frac{{ℜ}_{s}}{KM}$$

**Figure 2:**
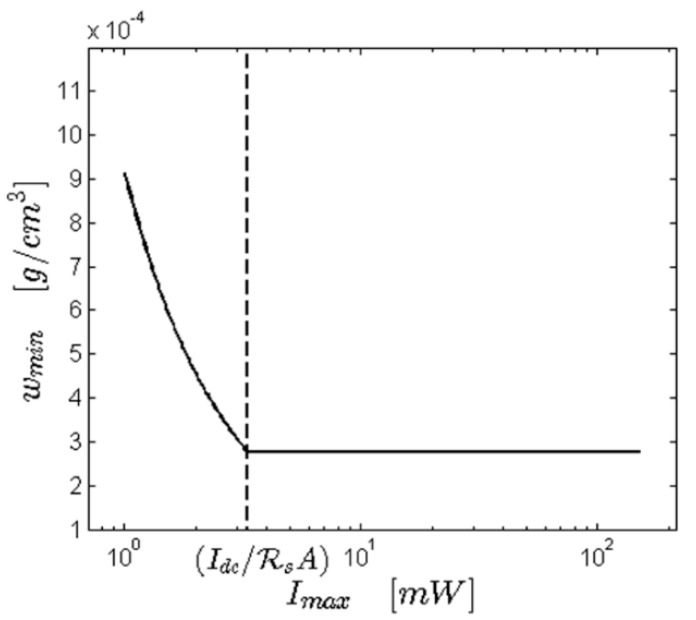
Minimum measurable particle concentration as a function of the maximum available laser power for the case of lysozyme and instrument sensitivity as detailed in Section 4.3. The vertical dashed line marks the boundary between the two regimes as detailed in the text.

The DLS correlation function’s intercept *β* is a measure of the signal-to-noise ratio, and for concentrations approaching $w_{\min}$ the intercept will gradually approach zero. This can be intuitively understood from Equation (5) by noting that for *β* = 0 the diffusion coefficient can no longer be inferred form the measured correlation function. In this case, the maximum laser power affects the duration of the measurement required to achieve a certain accuracy. More precisely, for particle concentrations close to the value in Equation (9) one might obtain DLS measurements with low intercepts, if the concentration is too low, the required measurement time to achieve a reasonable accuracy might be prohibitively long. In such cases, a stronger maximum laser power will help reducing the measurement time and consequently widen the measurability range, given that the intercept is sufficiently high to avoid an *S*/*N* ratio that is effectively zero. Figure 2 summarizes these considerations by plotting the minimum measurable concentration as a function of the maximum available laser power. Please note that for *I*_max_ lower than *I_DC_*/(ℜ*_s_A*), *w*_min_ follows the law in Equation (8), while for larger laser intensities it settles to the constant value according to Equation (9). One can conclude that a DLS instrument should ideally deliver a laser power larger than the threshold value *I_DC_*/(ℜ*_s_A*) of the solvents that the experimentalist plans to routinely measure. Once this is ensured, even larger laser power may lower the effective lowest measurable concentration.

### 4.2. Sensitivity in Protein Solution Measurement: Theoretical Approach

Proteins have different sizes that are often around or even below 10 nm, which is close to the lower end of DLS size measurability range. Since, as shown in Equation (1), the scattered intensity is proportional to the product of the mass concentration times the molar mass, it is generally challenging to measure them in dilute conditions. Particularly difficult to measure is the comparatively small model protein lysozyme (14.3 KDa) that was selected as model for the current study on laser power effects in DLS. Since there is an interest in *w*_min_, it must be clarified whether the signal arising from the solvent is substantially larger than that of the dark counts (Equation (9)) or if the case is given where signal originating from the scattering by the solvent at the maximum available laser power is not substantially larger than that of the detector dark counts (Equation (8)). Therefore, the intensity scattered by water (*I_w_*) has to be calculated in order to determine whether the scattering of the solvent is significantly larger than that of the dark counts. As in Equation (10) (here the value for *I*_0_ represents the available power in an instrument equipped with a 100 mW laser), the scattered intensity of the water can be calculated by knowing both ${ℜ}_{w}$ and *A*. The Rayleigh ratio of water is reported in the literature [20] as ${ℜ}_{w}=0.92\cdot 10^{-6}\;{\mathrm{cm}}^{-1}$. The instrument sensitivity constant *A* for a pure solvent can be determined from the reported Equation (10).

(10) $$I_{\mathrm{ref}}=I_{0}A{ℜ}_{\mathrm{ref}}$$

When the solvent’s Rayleigh ratio is known (at a certain temperature and wavelength corresponding to the one adopted in the instrument), *A* can be calculated from the intensity scattered by the solvent at a given input power (see Equation (11)).

(11) $$A=\frac{I_{\mathrm{ref}}}{I_{0}{ℜ}_{\mathrm{ref}}}$$

The Rayleigh ratio values have been tabulated for several common solvents, the most common being toluene, given its relatively high value of the Rayleigh ratio. A recent publication reports an empirical relationship [21], derived from all the available experimental data collected for toluene, that allows calculation of toluene Rayleigh ratios for vertically polarized incident light at a given wavelength. The expression is as follows:

(12) $${ℜ}_{\mathrm{tol}}=\frac{2\cdot 4.90\;{\mathrm{cm}}^{-1}\cdot 10^{6}{\left(\lambda /\mathrm{nm}\right)}^{-4.17}}{1+0.492}$$

This equation is valid for the full range 400–700 nm at 25 °C. It provides accurate estimates to within 6% at λ=532 nm. In the case of the instrument we used (NanoLab 3D), equipped with a 660 nm, 100 mW, vertically polarized laser, with the detector at 90° with respect to the incident beam, the instrument constant *A* was determined as follows. A set of 10 measurements of 10 s each yielded an average detected light power normalized to the incident laser power (*I*_ref_/*I*_0_) of 3.8 kHz/mW. With the Rayleigh ratio of toluene at 660 nm ${ℜ}_{\mathrm{Tol}}=1.14\times 10^{-5}\;{\mathrm{cm}}^{-1}$, the given instrument constant is $A=3.31\times 10^{5}\;\frac{\mathrm{kHz}}{\mathrm{mW}}\cdot \mathrm{cm}$. By using these calculated values, the intensity scattered by water (*I*_w_) is:

(13) $$I_{w}=\left(75\;\mathrm{mW}\right)\left(3.31\times 10^{5}\frac{\mathrm{kHz}}{\mathrm{mW}}\cdot \mathrm{cm}\right)\left(0.92\times 10^{-6}\;{\mathrm{cm}}^{-1}\right)=22.8\;\mathrm{kHz}$$

This value is above the typical detector dark counts, which typically fall below 1 kHz. Having established that we are in the regime in which the signal arising from the solvent is substantially larger than that of the dark counts ($w_{\min}=\frac{{ℜ}_{s}}{\mathrm{KM}}$), in order to calculate $w_{\min}$, one needs to evaluate the constant K using the following equation (Equation (14))

(14) $$K=\frac{{\left[2\pi n_{s}\right]}^{2}}{N_{A}\lambda^{4}}{\left[\frac{dn}{dc}\right]}^{2}$$

where *n_s_* is the refractive index of the pure solvent, *dn*/*dc* is the refractive index increment of the solute/solvent system, λ is the wavelength of the laser used and $N_{A}$ = 6.022 × 10^23^ mol^−1^ is the Avogadro’s number. The refractive index increment is a measure of the optical contrast between the solute molecules and the solvent. This can be determined by measurement of the refractive index of solutions at different concentrations of the solute molecules. Often, tabulated values are used instead of measured ones. For lysozyme (14,307 Da), one gets *dn/dc* = 0.194 mL/g [22].

Having all the parameters to fill Equation (9), it is possible to calculate the lysozyme minimum concentration, $w_{\min}$ to be $2.78\times 10^{-4}\;\frac{g}{{\mathrm{cm}}^{3}}$. It should be noted that the above calculation assumes that all the available laser power indeed reaches the sample. For a comparison, similar calculations were performed for an instrument equipped with a 45 mW laser source at 685 nm. After computing the contrast for this wavelength and considering an instrument with similar sensitivity (same instrument constant), with the available power at 15 mW, our calculations yield intensities of I ~ 4.6 kHz for the scattering signal arising from water, which is only about 4 times the value of the dark counts (1 kHz) and it cannot be considered substantially larger than that of the dark counts. Our criterion to be considered as such is that of *I*/*I*_DC_ > 10.

### 4.3. Sensitivity in Protein Solution Measurement: Experimental Approach

This section approaches the topic of laser power in DLS from an experimental perspective. In line with the difficulty of measuring proteins of around or below 10 nm in dilute colloidal dispersion, lysozyme was selected as the model system. In a dilute aqueous condition, sample preparation can represent one of the major obstacles to obtain accurate size measurements. The sample must be free of particulate impurities (such as dust particles) because of the low amount of light scattered by lysozyme. Moreover, in highly dilute conditions, protein aggregation can suppress or hide the scattering signal arising from the monomers [23,24]. Scattering intensity is proportional to *R_h_*^6^; therefore, the presence of trace amounts of aggregate is easily detectable. Moreover, the presence of aggregates will result in a significant change in the mean hydrodynamic radius and in the polydispersity index. In the particular case of lysozyme, aggregation may occur in a time scale of minutes, and even small traces of aggregates may skew the measurements [13]. For these reasons, the sample must be handled under a laminar flow hood to avoid contamination, and nanofiltration (e.g., using a 20 nm Anatop filter) is recommended to obtain a size estimate of non-aggregated protein, with each measurement carried out immediately after filtration.

**Figure 3:**
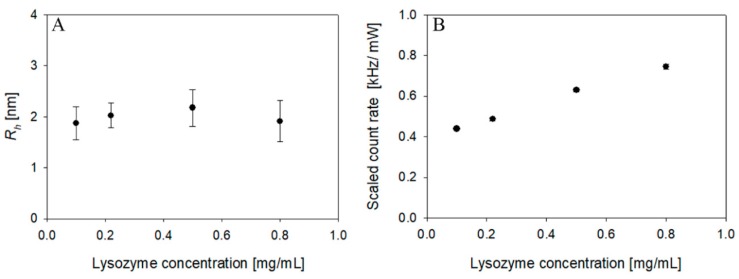
Hydrodynamic radius (*R_h_*) (**A**) and scaled count rate (**B**) of lysozyme at different concentration (100 mW laser intensity).

**Figure 4:**
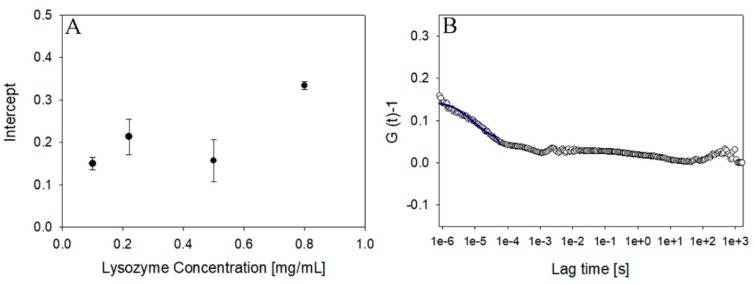
Intercept of lysozyme at different concentrations (**A**) and correlation function of lysozyme at lowest detectable concentration (**B**) (0.1 mg/mL, 100 mW laser intensity).

The present study considered two different laser intensities as part of separate instruments. In the case of 45 mW laser power, the detection limit of the instrument was reached with a lysozyme concentration of 2 mg/ mL (see Table 1). At this concentration, the intercept (0.2 ± 0.01) and the scaled count rate (3.2 ± 0.05) were still acceptable to determinate the size of the protein (2.04 ± 0.2 nm) using the cumulant method. Decreasing the concentration to 1 mg/mL resulted in the intercept dropping to zero, rendering it impossible to determine the hydrodynamic radius. On the other hand, as reported in Figure 3, using a nominal power laser intensity of 100 mW, it is possible to measure the hydrodynamic radius, down to a 0.1 mg/mL lysozyme dispersion. Even in these diluted conditions, and thanks to the sample filtration, there were no traces of aggregates and the hydrodynamic radius (*R_h_*) of lysozyme evaluated according to the cumulant method was 1.9 ± 0.3 nm, which is in agreement with the value reported in literature [25]. Interestingly, as shown in Figure 4, the intercept even at this highly diluted condition had a value of 0.15, which was still acceptable for size determination.

## 5. Conclusions

DLS is of substantial importance for measurement of biopharmaceutical formulations and other colloidal dispersions. The present work addressed an important knowledge gap to study effects of laser intensity on the sensitivity of DLS instruments and subsequent quality of DLS size measurements. Depending on the experimental samples, the light scattered by the solvent may either be substantially higher than the dark counts of the detector or below this threshold. Consequences of these two scenarios were first considered theoretically and equations were given to estimate sample concentration limit $w_{\min}$ depending on the given laser intensity. For the experimental part, lysozyme was chosen as the model drug, since determining its size by DLS is challenging in highly diluted aqueous solutions. Two comparable instruments with different laser intensities (45 and 100 mW) were tested and the higher laser intensity was found to be advantageous to measure at high dilution in a more robust way. A minimum measurable particle concentration of 0.1 mg/mL was achieved at 100 mW, which was of the same order of magnitude as expected theoretically. Important was that the higher laser power enabled measurement of such low concentration of lysozyme with an experimental protocol that was still practical from an industrial viewpoint. It is shown, then, that a higher laser intensity aids in reducing the measurement time, as well as the minimum concentration measurable by the instruments. The limits for the maximum applicable laser power are dependent on the absorption properties specific to the systems to be studied. High intensity may lead to local heating and apparent smaller measured hydrodynamic radii, if the absorption is considerable. The present work also outlined different experimental details that affect size measurement to enable a meaningful comparison of any instrumental factor. This is an important aspect when application scientists compare specifications of DLS instruments. Given the light scattering characteristics of typical samples, a suitable analytical instrument with sufficient laser intensity should be selected. Current findings provide guidance not only regarding laser intensity but also regarding the definition of viable experimental protocols for adequate size experiments by DLS, particularly required in analytics of biopharmaceutical medicines, as well as to measure other colloidal systems. 

## Figures and Tables

**Table 1:** Intercept, scaled count rate and hydrodynamic radius (*R_h_*) of lysozyme at different concentrations obtained with the 45 mW laser power.

Concentration (mg/mL)	Intercept	Scaled Count Rate (kHz/mW)	*R_h_* (nm)
5	0.40 ± 0.04	6.2 ± 0.02	1.94 ± 0.4
3	0.38 ± 0.02	5.2 ± 0.08	2.20 ± 0.3
2	0.20 ± 0.01	3.2 ± 0.05	2.04 ± 0.2
1	N.A *	N.A *	N.A *
0.8	N.A *	N.A *	N.A *
0.5	N.A *	N.A *	N.A *

* Measurement not Available.
